# Machine Learning Based Abnormal Gait Classification with IMU Considering Joint Impairment

**DOI:** 10.3390/s24175571

**Published:** 2024-08-28

**Authors:** Soree Hwang, Jongman Kim, Sumin Yang, Hyuk-June Moon, Kyung-Hee Cho, Inchan Youn, Joon-Kyung Sung, Sungmin Han

**Affiliations:** 1 Bionics Research Center, Biomedical Research Division, Korea Institute of Science and Technology (KIST), Seoul 02792, Republic of Korea; srhwang@kist.re.kr (S.H.); jmkim0127@kist.re.kr (J.K.); s.yang@kist.re.kr (S.Y.); crescent@kist.re.kr (H.-J.M.); iyoun@kist.re.kr (I.Y.); 2 School of Biomedical Engineering, Korea University, Seoul 02841, Republic of Korea; 3 Department of Neurology, Korea University Anam Hospital, Korea University College of Medicine, Seoul 02841, Republic of Korea; bluedoc76@naver.com; 4 Division of Bio-Medical Science & Technology, KIST School, Korea University of Science and Technology, Seoul 02792, Republic of Korea; 5 KHU-KIST Department of Converging Science and Technology, Kyung Hee University, Seoul 02447, Republic of Korea

**Keywords:** abnormal gait, joint impairment, IMU-based system, walkway system, RFECV, machine learning classification

## Abstract

Gait analysis systems are critical for assessing motor function in rehabilitation and elderly care. This study aimed to develop and optimize an abnormal gait classification algorithm considering joint impairments using inertial measurement units (IMUs) and walkway systems. Ten healthy male participants simulated normal walking, walking with knee impairment, and walking with ankle impairment under three conditions: without joint braces, with a knee brace, and with an ankle brace. Based on these simulated gaits, we developed classification models: distinguishing abnormal gait due to joint impairments, identifying specific joint disorders, and a combined model for both tasks. Recursive Feature Elimination with Cross-Validation (RFECV) was used for feature extraction, and models were fine-tuned using support vector machine (SVM), random forest (RF), and extreme gradient boosting (XGB). The IMU-based system achieved over 91% accuracy in classifying the three types of gait. In contrast, the walkway system achieved less than 77% accuracy in classifying the three types of gait, primarily due to high misclassification rates between knee and ankle joint impairments. The IMU-based system shows promise for accurate gait assessment in patients with joint impairments, suggesting future research for clinical application improvements in rehabilitation and patient management.

## 1. Introduction

Walking is the most fundamental movement for maintaining a healthy daily life and is essential for performing a variety of purposeful activities. Normal walking requires the coordinated action of the central and peripheral nervous systems, as well as the musculoskeletal system. However, numerous abnormal gait patterns can arise because of various factors, including the weakening of the musculoskeletal and nervous systems due to aging, diseases, accidents, and post-surgical complications [1]. Stroke patients often exhibit joint stiffness and hemiparetic gait due to lesions in the cerebral hemisphere or ataxic gait and impaired balance due to cerebellar lesions [2]. Patients with Parkinson’s disease may experience difficulty initiating walking and problems with turning, avoiding obstacles, and stopping due to bradykinesia, rigidity, and postural instability associated with extrapyramidal system dysfunction [3]. Additionally, patients with cerebral palsy suffered diplegic gait and crouch gait due to the loss of neuromuscular control, making it difficult to maintain posture [4]. These abnormal gait patterns lead to issues of balance and stability, pain and discomfort, limited mobility, complex daily management, and significant psychological effects, all of which collectively deteriorate the overall quality of life.

For these reasons, many researchers have been studying the causes of abnormal gait and simultaneously evaluating the types and severity of diseases, as well as the effectiveness of rehabilitation training, through gait analysis [5]. In particular, with the increase in joint surgery patients due to advancements in artificial joint technology [6], the importance of monitoring the prognosis and analyzing the effects of rehabilitation exercises in patients who are unable to walk normally due to joint surgery or paralysis has grown. Consequently, there has been active research on analyzing lower limb joint kinematics, determining abnormal gait, and classifying walking conditions [7]. To detect and classify abnormal gait, conventional methods frequently use marker-based motion capture systems. These systems are known for their high accuracy but come with significant drawbacks: they are expensive, require complex setups, and depend on skilled evaluators [8]. As a result, their use is often limited to large institutions, making them impractical for routine clinical use and leading to delays in diagnosis and treatment.

In response to these limitations, more accessible and portable gait measurement devices, such as the walkway system, have been developed. The walkway system, which uses pressure sensors, is commonly employed in clinical and research settings due to its ability to accurately measure forces and pressures exerted on the feet during walking [9]. Webster et al. conducted a gait analysis study on elderly patients who underwent knee arthroplasty using both the electronic walkway system (GAITRite^®^, GAITRite Gold, CIR Systems, Havertown, PA, USA) and the 3D motion analysis system (Vicon-512^®^, Oxford Metrics, Oxford, UK) by calculating gait parameters [10]. The results demonstrated that the walkway system showed high intra-class correlation coefficients (0.92 to 0.99) and excellent repeatability coefficients (1.0% to 5.9%) compared to the 3D motion analysis system, thus validating the effectiveness of the walkway for gait analysis. Similarly, Stokic et al. evaluated spatio-temporal gait parameters in healthy individuals and chronic stroke patients using the walkway and the 3D motion analysis system (EVaRT) [11]. They reported that the walkway system performed as well as the 3D motion analysis system. However, it requires ample space for installation and can measure foot forces only, which can make it difficult to capture joint angle deviations observed in asymmetric gait [12]. Moreover, the cost of acquiring and maintaining force plates can be prohibitive.

**Figure 1:**
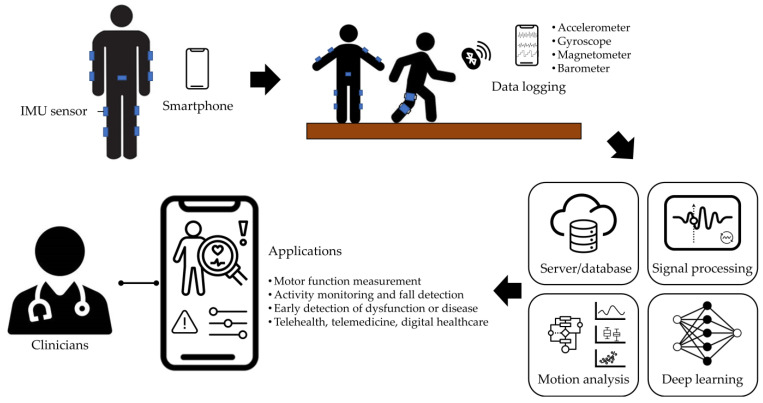
An example of an IMU-based healthcare platform.

To overcome these issues, numerous studies utilizing wearable sensors have been proposed, among which research employing inertial measurement unit (IMU) has reported high usability and excellent performance [13,14]. These compact and portable sensors are suitable for use in real-world environments and can measure a wide range of movements and postures. While they may offer lower accuracy compared to force plates, precise sensor placement and calibration can mitigate these discrepancies. IMUs can directly measure joint angles and extract general gait parameters, making them useful for analyzing asymmetric gait. Wang et al. used IMUs attached to the left and right shanks to analyze the gait of healthy adults and patients with neuropathy-induced abnormal gait, and they reported that IMUs, as well as 3D motion analysis systems (Vicon-T40S), are suitable for gait analysis in both healthy individuals and patients [15]. In another study, IMUs and a deep neural network algorithm were used to detect abnormal gait and classify four gait patterns in stroke patients [16]. Numerous studies have validated the accuracy of IMU measurements, demonstrating their reliability in capturing general gait parameters and joint angles [17]. Based on these research findings, IMU-based healthcare platforms, which utilize IMU systems integrated with smartphones and network servers to record and analyze movements in real-time, were recently proposed for the assessment of users’ motor functions and digital healthcare (Figure 1).

This study aims to investigate whether an IMU-based system can effectively classify abnormal gait patterns caused by knee and ankle joint impairments. While previous research has compared the accuracy of specific gait parameters between the IMU-based and walkway systems, this study focuses on evaluating the capability of IMUs to detect and classify gait abnormalities specifically related to joint impairments. Walking measurements were conducted using both systems on the same participants to assess the performance of IMUs in a clinical setting. Through this comparison, this study aims to determine the effectiveness of IMUs in providing accurate and reliable gait assessments for patients with joint impairments.

## 2. Materials and Methods

### 2.1. Participant Recruitment

Prior to participation, written consent was obtained from all subjects, following the guidelines set by the Institutional Review Board of the Korea Institute of Science and Technology (IRB No. 2022-002). This study involved ten healthy male participants. The participants had an average age of 39.4 ± 10.3 years, an average height of 175.6 ± 6.1 cm, and an average weight of 79.0 ± 12.6 kg (Table 1). All subjects were required to have had no special neurological or orthopedic abnormal findings within the last 5 years.

### 2.2. Experimental Instrumentation and Procedure

**Figure 2:**
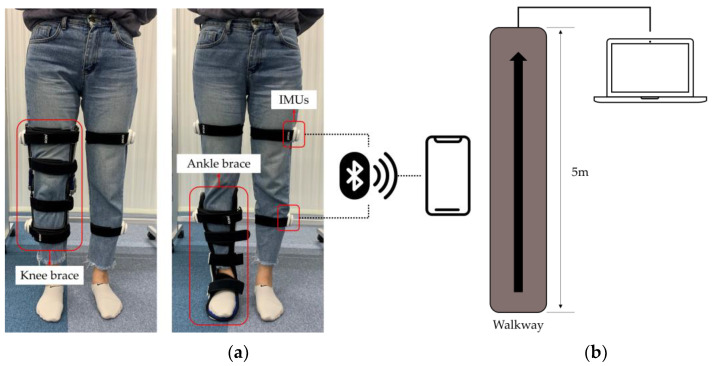
Experimental setup for collecting gait data from joint impairment walking. (**a**) IMU-based system with wearable braces: knee brace (**left**) and ankle brace (**right**) equipped with IMUs. (**b**) Five-meter walkway system for gait analysis.

Braces can constrain knee and ankle kinematics and reduce load and force on the affected compartments. Participants simulated joint impairments by wearing a knee brace (ACL, Medicalnine Inc., Seoul, Korea) on the right leg or an ankle brace (Nice stretch 90, Brownmed Inc., Boston, MA, USA) on the right leg to restrict joint movement. While walking, kinematics data were acquired using both an IMU-based system (eCEN care, eCEN Inc., Seoul, Korea) and a walkway system (GAITRite^®^, CIR Systems Inc., Franklin, NJ, USA), simultaneously. The IMU sensor includes 3-axis accelerometers, 3-axis gyroscopes, and a Bluetooth 4.0 module. Each IMU measured 43 mm × 41.86 mm × 18.65 mm and weighed 12 g, including the power source. Data were collected at a sampling frequency of 50 Hz and transmitted up to 10 m. The IMU sensor was attached to both lateral thighs and shanks parallel to the sagittal plane (Figure 2). The data collected from the four IMU sensors were processed internally to include Euler rotation angles and exported in .csv file format.

The walkway system featured a matrix of pressure-activated sensors for detecting timing and relative distances between activations. With dimensions of 5 m in length and an active measurement range of 3 m, the walkway offered a spatial resolution of 1.27 cm (determined by the length/width of sensors) and operated at a temporal resolution of 120 Hz. The data collected from the walkway system were processed internally to include gait parameters and exported in .csv file format.

All participants walked a straight 5 m path under three conditions: without wearing any joint braces, with a knee joint brace, and with an ankle joint brace. These conditions were designed to simulate three types of gaits: normal walking, walking with knee joint impairment, and walking with ankle joint impairment. They completed 10 trials at self-selected speeds under each condition.

### 2.3. Data Preprocessing

#### 2.3.1. Gait Event Detection

**Figure 3:**
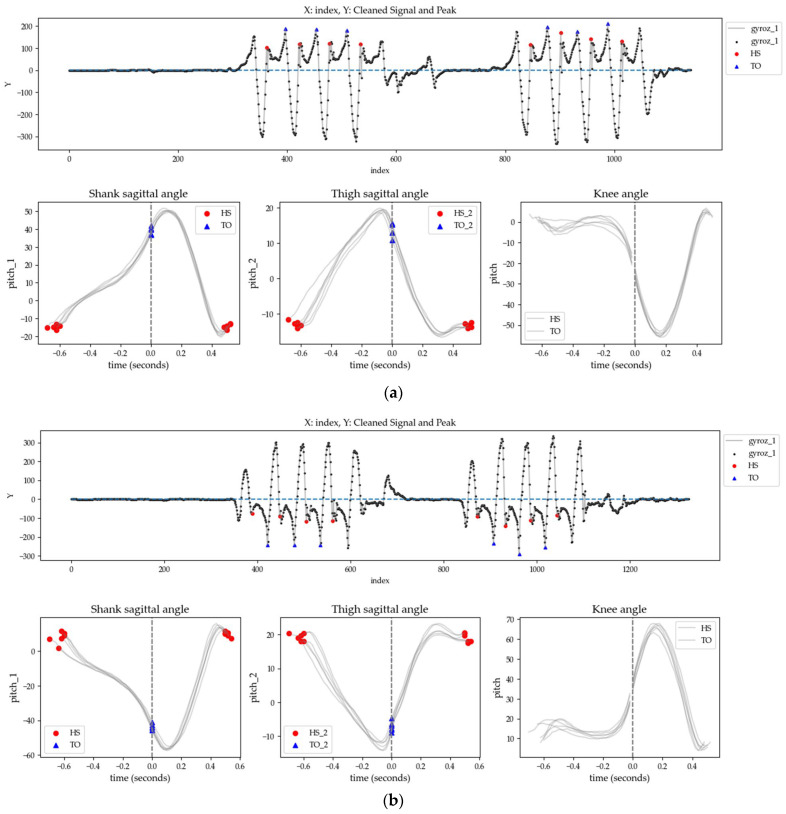
Gait events detected from shank angular rate during walking: the red dots represent HS points, and the blue triangles indicate TO points. Left and right steps are paired together. (**a**) The signals from IMUs attached to the left leg; (**b**) the signals from IMUs attached to the right leg.

The detection of gait events, such as heel-strikes (HS) and toe-offs (TO), was performed to extract gait parameters by gait cycle. HS and TO events were detected on the sagittal plane angular velocity of the shank IMU using the ‘find_peaks’ function from the SciPy library with prominence of 20 degrees per second and distance of 20 samples. The automated event detection results were verified through a final inspection process to ensure accuracy and minimize errors. The period between HS and the next TO was defined as the stance phase, while the period between TO and the next HS was defined as the swing phase. Each period from one HS to the next constituted a complete gait cycle. Gait cycles from both legs were paired based on overlapping time frames (Figure 3).

#### 2.3.2. Gait Parameter Extraction

Gait parameters encompassed angular, spatio-temporal, and symmetry parameters. The angular parameters, such as shank angle (deg), thigh angle (deg), and the range of knee joint angle (deg) in the sagittal plane, were calculated as the difference between maximum and minimum values. The spatio-temporal parameters, such as cycle time (s), cadence (steps/min), swing time (s), stance time (s), swing phase, and stance phase, were derived from the intervals between gait events. Cycle time is the duration between the first and the second HS. Cadence is calculated as the number of steps per minute derived from cycle time. Stance time is the duration between the first HS and TO, and swing time is the duration between the second HS and TO. Swing phase is the ratio of swing time to cycle time, and stance phase is the ratio of stance time to cycle time.

The symmetry parameters, such as the temporal symmetry ratio (TSR), the spatial symmetry ratio 1 (SSR 1), and the spatial symmetry ratio 2 (SSR 2), were calculated as the ratio of corresponding gait parameters between both legs. TSR compares the ratio of swing time to stance time between the right and left legs (Equation (1)). SSR 1 compares the ratio of the shank angle to the thigh angle between the right and left legs (Equation (2)), and SSR 2 compares the ratio of the knee joint angle between the right and left legs (Equation (3)). Excluding the symmetry parameters, the left and right parameters were calculated separately, resulting in a total of 21 parameters being extracted (Table 2).

(1) $$TSR=\frac{Swin{g\;time}_{\;R}/Stanc{e\;time}_{R}}{Swin{g\;time}_{L}/Stanc{e\;time}_{L}}$$

(2) $$SSR\;1=\frac{Shank\;angle_{R}/Thigh\;angle_{R}}{Shank\;angle_{L}/Thigh\;angle_{L}}$$

(3) $$SSR\;2=\frac{{Knee\;joint\;angle}_{R}}{{Knee\;joint\;angle}_{L}}$$

The walkway system provides a comprehensive set of spatio-temporal gait parameters, including step length (cm), stride length (cm), base of support (cm), step time (s), stride time (s), swing time (s), stance time (s), single support time (s), double support time (s), stride velocity (cm/s), swing phase, and stance phase. In this study, these parameters were used as calculated and provided by the system. TSR were calculated according to Equation (1). Excluding the symmetry parameters, the left and right parameters were calculated separately, resulting in a total of 25 parameters being extracted (Table 2).

### 2.4. Feature Selection and Classification

#### 2.4.1. Feature Selection

**Figure 4:**
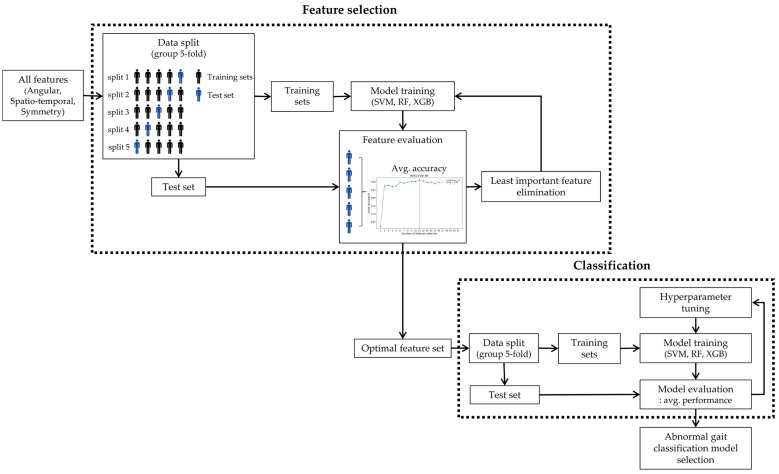
ML modeling framework for abnormal gait classification.

Feature selection plays a crucial role in classification tasks by eliminating irrelevant information from models, thereby enhancing efficiency and accuracy. In this study, we systematically evaluated feature importance and identified the optimal subsets using Recursive Feature Elimination with Cross-Validation (RFECV). We divided the gait data into five subject-based subsets using group five-fold cross-validation, designating one subset as the test set and utilizing the remaining four for training. The models were trained sequentially, iteratively eliminating the least significant features. In this study, three different methods, support vector machine (SVM), random forest (RF), and extreme gradient boosting (XGB), were used, and the optimal feature set was selected based on the average accuracy across all folds (Figure 4).

#### 2.4.2. Classification

The primary objective of this study is to evaluate whether IMU-based systems can effectively detect and classify abnormal gait caused by knee and ankle joint impairments. To achieve this, different types of gait, such as normal, knee joint brace, and ankle joint brace gaits, were classified using three types of classifiers. SVM handles both linear and nonlinear tasks, identifying the optimal hyperplane that effectively separates data points. The SVM configuration used a ‘linear’ kernel, with gamma set to ‘scale’ and the decision function shape set to ‘ovr’. RF combines decision trees to provide accurate predictions and utilizes parallel processing for faster computation. The RF configuration used the ‘gini’ criterion with a minimum sample split of 2. XGB constructs decision trees sequentially to correct errors, ensuring efficient processing even with large datasets. For XGB, the booster was set to ‘gbtree’, eta to 0.3, maximum depth to 6, and sampling method to ‘uniform’; the objective to minimize regression error. All analyses were performed using Python version 3.10.9.

To develop the models, we trained and evaluated them using the optimal features selected through RFECV. The gait data were divided into five subject-based subsets using group five-fold cross-validation. Each subset was alternately designated as the test set while the remaining four subsets were used for training. After performing hyperparameter tuning to optimize the model parameters for better performance, we used the optimized model to make predictions on the test set and evaluated its performance using metrics such as accuracy, precision, recall, and F1 score (Figure 4). These metrics were computed using Equation (4) through (7), where TP, TN, FP, and FN represent True Positive, True Negative, False Positive, and False Negative, respectively.

(4) $$Accuracy=\frac{TP+TN}{TP+FP+TN+FN}$$

(5) $$Precision=\frac{TP}{TP+FP}$$

(6) $$Recall\;(Sensitivity)=\frac{TP}{TP+FN}$$

(7) $$F1\;score=2\times \frac{Precision\times Recall}{Precision+Recall}$$

#### 2.4.3. Post-Analysis

In the post-analysis stage, we examined the differences in selected features across three different gait types using the Friedman test. Subsequently, pair-wise comparisons were conducted using the paired Wilcoxon signed-rank test to investigate differences in selected features between pairs of gait types. The significance levels were set to 0.05* and 0.01**. Bonferroni correction was applied to control the family-wise error rate, and the significance levels were divided by the number of comparisons to adjust for multiple comparisons.

## 3. Results

This section presents performance metrics for classification models based on optimal features. Table 3 shows the results of learning and evaluation with selected features based on the RFECV method.

In the IMU-based system, the optimal number of selected features ranged from 1 to 18 out of a total of 21 parameters, depending on the specific machine learning model employed. For the normal and abnormal classification, the RF classifier achieved 99% in all performance metrics using 12 optimal features. For the normal and knee joint impairment classification, the SVM and RF classifier achieved 100% in all performance metrics using one optimal feature. In the case of the normal and ankle joint impairment classification, the RF classifier achieved 98% precision and 97% in all other performance metrics using two optimal features. For the knee joint impairment and ankle joint impairment classification, the RF classifier and XGB classifier achieved 98% in all performance metrics using 18 and 3 optimal features, respectively. For the normal, knee joint impairment, and ankle joint impairment classification, the RF classifier achieved 91% in all performance metrics using 11 optimal features. The three-class classification used more optimal features than the two-class classification models yet achieved lower accuracy.

In the walkway system, the optimal selection of features ranged from 1 to 21 out of a total of 25 parameters, depending on the specific machine learning model employed.

For the normal and abnormal classification, the SVM classifier achieved 90% accuracy and recall, 92% precision, and 91% F1 score using one optimal feature. For the normal and knee joint impairment classification, the RF classifier achieved 93% F1 score and 94% in all other performance metrics using three optimal features. In the case of the normal and ankle joint impairment classification, the SVM classifier achieved 91% in all performance metrics using 12 optimal features. For the knee joint impairment and ankle joint impairment classification, the SVM classifier achieved 76% in all performance metrics using nine optimal features. For the normal, knee joint impairment, and ankle joint impairment classification, the XGB classifier achieved 77% in all performance metrics using 21 optimal features.

Table 4 shows the common optimal features of all classifiers in gait classification. Key features of the IMU-based system include TSR, the knee sagittal angle of the right leg, and shank sagittal angle of the right leg. Meanwhile, key features of the walkway system include TSR, swing time of the right leg, stance time of both legs, swing phase of both legs, and double support time of both legs.

**Figure 5:**
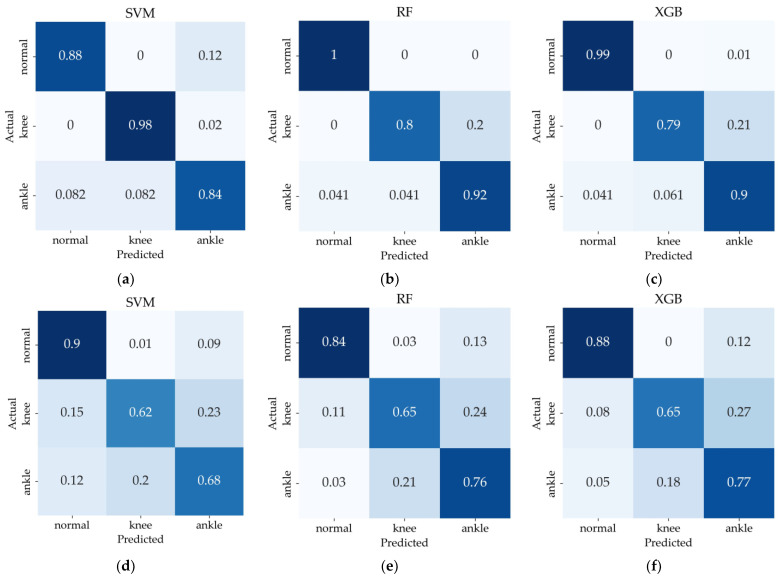
Confusion matrix in validation dataset of normal vs. knee joint impairment vs. ankle joint impairment: (**a**–**c**) Classification results of IMU-based system, (**d**–**f**) Classification results of walkway system. (**a**,**d**) SVM, (**b**,**e**) RF, (**c**,**f**) XGB models. Darker shades indicate a greater number of correctly classified instances for each category.

Figure 5 shows the confusion matrix for classification of the normal, knee joint impairment, and ankle joint impairment. In the IMU-based system, the RF classifier achieved the highest recall for the normal at 100%, whereas the SVM classifier achieved the lowest at 88%. For the knee joint impairment, the SVM classifier achieved the highest recall at 98%, while the XGB classifier achieved the lowest at 79%. In the ankle joint impairment, the RF classifier achieved the highest recall at 92%, whereas the SVM classifier achieved the lowest at 84%.

In the walkway system, the recall performance for the normal was notably high, ranging from 84% to 90% across all classifiers. However, the highest recall performance for the knee joint impairment, achieved using the RF and XGB classifiers, was 65%, which was significantly lower compared to the normal. The misclassification rates into the ankle joint impairment for knee joint impairment were 24% for the RF classifier and 27% for the XGB classifier, in contrast to misclassification rates into the normal of less than 5%.

Furthermore, the highest recall performance for the ankle joint impairment, achieved with the XGB classifier at 77%, was also notably lower compared to the normal. The misclassification rate into the knee joint impairment for the ankle joint impairment was 18%, higher than the 8% misclassification rate into the normal.

It appears that the parameters provided by the walkway system do not effectively capture the distinguishing features between knee joint impairment walking and ankle joint impairment walking, resulting in a higher misclassification rate. This insight helps to explain why the highest performance in the three-class classification reached only 77%.

Table 5 presents the performance metrics from the 5-fold cross-validation of the highest-performing classifiers in the IMU-based system and the walkway system, respectively. The RF classifier in the IMU-based system exhibited high performance in Fold 2 and lower performance in Fold 3, and it maintained excellent performance overall. In contrast, within the walkway system, the XGB classifier performed well in Fold 1 but showed lower performance in Fold 3, demonstrating relatively lower performance compared to the IMU-based system. These 5-fold cross-validation results, which were used to evaluate model performance and minimize the risk of overfitting, indicate that the IMU-based system with the RF classifier not only outperforms but also exhibits lower performance variability compared to the walkway system with the XGB classifier.

We further analyzed the optimal features selected for walking parameters to identify characteristic differences among three different types of gait. Table 6 presents the mean and standard deviation of each parameter for each condition, along with the results of the Friedman test to determine differences in each parameter under different gait conditions. The results showed no significant differences among groups for the thigh sagittal angle, swing time of the left leg, and single support time of the right leg, with *p*-values being above 0.05. However, all other parameters exhibited significant differences.

**Figure 6:**
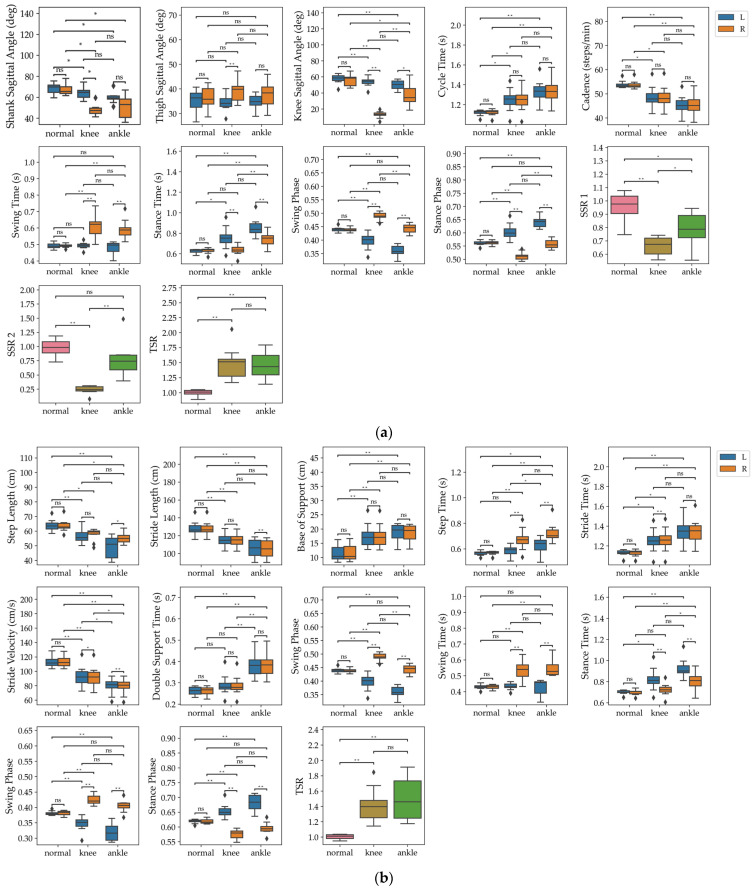
Box plot and Wilcoxon signed-rank test results of gait parameters measured by (**a**) the IMU-based system and (**b**) the walkway system in normal walking, walking with knee joint impairment, and walking with ankle joint impairment. Significance levels are set at * *p* < 0.05 (indicated by *), ** *p* < 0.01 (indicated by **), and ns (not significant).

To identify which groups had significant differences among the parameters with detected differences, we performed a post hoc analysis using the paired Wilcoxon signed-rank test to compare the two conditions within the same subjects (Figure 6).

The analysis revealed several noteworthy observations. In the IMU-based system, knee joint impairment exhibited significant differences between legs for the shank sagittal angle, thigh sagittal angle, knee sagittal angle, swing time, stance time, swing phase, and stance phase within each condition. Specifically, the parameters of the unrestricted leg, such as the shank sagittal angle and knee sagittal angle, showed a slight decrease, while those of the restricted leg significantly decreased. Our findings are consistent with previous studies indicating that wearing a knee brace reduces maximum internal knee rotation and increases external rotation [18]. Moreover, swing phase tended to decrease in the unrestricted leg and increase in the restricted leg. With ankle joint impairment, although the differences were less pronounced compared to knee joint impairment, significant differences were observed between legs in the shank sagittal angle, knee sagittal angle, swing time, stance time, swing phase, and stance phase within each condition. Similar to knee joint impairment, the shank sagittal angle and knee sagittal angle significantly decreased in both legs compared to the normal condition. Also, the swing phase tended to decrease in the unrestricted leg and increase in the restricted leg. SSR 1 and TSR indicated increased asymmetry in abnormal walking. SSR 1 and SSR 2 showed greater asymmetry in knee joint impairment compared to ankle impairment, while TSR did not show any differences between knee and ankle joint impairment (Figure 6a).

In the walkway system, several differences were observed between normal and abnormal walking. In abnormal walking, both legs showed decreases in step length, stride length, and stride velocity, and increases in base of support and stride time. Single support time increased on the unrestricted side, while swing time increased on the restricted side. In knee joint impairment, the swing phase decreased on the unrestricted side and increased on the restricted side. Double support time significantly increased only during ankle joint impairment walking. The TSR showed increased asymmetry in abnormal walking, but no significant differences were found between knee and ankle joint impairments (Figure 6b).

## 4. Discussion

In this study, we developed and optimized an abnormal gait classification algorithm that considers joint impairment using IMU-based and walkway systems. The results demonstrated that the IMU-based system outperformed the walkway system in all classification tasks. Particularly noteworthy was the significant difference observed in the two-class classification tasks between knee joint impairment and ankle joint impairment (accuracy: IMU-based system 91% vs. walkway system 76%). This performance exceeds the accuracy reported in previous studies using IMU-based gait assessment for two-class patient classification (accuracy ranging from 76.3% to 89.3%) [19,20]. These findings indicate that our IMU-based system could be effective for classifying gait patterns with joint impairments.

For decades, various studies have been conducted to distinguish between pathological and normal gait, and more recently, machine learning-based gait pattern classification research has been reported for this purpose [21,22,23,24,25]. Alaqtash et al. used GRF (ground reaction force) signals to classify patients with cerebral palsy, multiple sclerosis, and healthy individuals, achieving an accuracy of 85% with a KNN (k-nearest neighbors) model [21]. Pogorelc et al. applied a motion capture system to extract 13 features from joint angles and spatio-temporal parameters, classifying individuals as normal, with hemiplegia, with Parkinson’s disease, with back pain, and with leg pain. They reported accuracy rates of 97.9%, 90.1%, 100%, 97.2%, and 100% using SVM, decision trees (DTs), k-nearest neighbors (KNN), naive Bayes (NB), and artificial neural networks (ANNs), respectively [23]. However, these studies have significant spatial limitations compared to wearable sensors like IMUs or footswitches, making them less practical. Conversely, Daliri used temporal gait parameters obtained from footswitches and an SVM to classify Parkinson’s disease, Huntington’s disease, multiple sclerosis, and healthy individuals, achieving accuracies of 89.3%, 90.3%, and 96.8%. However, this study did not perform intra-disease classification [25]. Consequently, our study demonstrates that IMU-based systems can be highly effective in classifying gait patterns with joint impairments across various contexts, potentially offering more practical and accurate assessments compared to traditional methods.

The superior classification performance of the IMU system compared to the walkway system can also be inferred from the optimal number of features presented in Table 3. In the IMU-based system, fewer features were used to achieve higher accuracy compared to the walkway system, excluding the cases of two-class classification for normal-ankle using XGB and three-class using RF classifiers. Particularly noteworthy is that distinguishing between normal and knee joint impairment was possible with just one feature: the knee sagittal angle. This ability stems from the fixed nature of the knee joint, making it a distinct and effective feature for classification.

Moreover, our study highlighted the importance of specific features in improving classification performance. Key features of the IMU-based system included the knee sagittal angle of the right leg, shank sagittal angle of the right leg, and TSR, providing the highly discriminative results. Angular parameters were particularly effective in distinguishing between knee and ankle joint impairments, as they capture subtle variations in joint movement that are not as easily detected with spatio-temporal parameters alone.

On the other hand, the walkway system relied more on spatio-temporal parameters such as stance time, swing phase, and double support time in both legs, swing time in the right leg, and TSR. While these features are useful for general gait analysis, they proved less effective in distinguishing specific joint impairments, as evidenced by lower classification accuracy in tasks such as knee joint impairment walking versus ankle joint impairment walking and in the three-class classification of gait types. These classification results are related to the previous study of Begg et al., which reported that basic spatial-temporal and kinetic gait features are not enough to classify the gait of young subjects and the elderly [24]. In a previous study, an SVM model using only kinematic data achieved a high classification accuracy of 91.7%, emphasizing the importance of kinematic features in gait classification. These results suggest that more specific features, such as kinematic data, are critical for classifying joint impairments rather than relying solely on spatio-temporal parameters.

Our statistical analysis further supported these findings. The detailed examination of walking parameters revealed significant differences in several key metrics under different joint impairment conditions. Particularly noteworthy was the heightened asymmetry observed in angular parameters during constrained walking, particularly evident in IMU-based systems. This underscores the sensitivity of angular measurements in identifying gait abnormalities.

However, this study has several limitations. The first limitation is the small sample size of participants because this study was performed as exploratory research focused on the evaluation of the validity and possibility of the proposed methodology at an early stage. Despite the small sample size, this study provides preliminary insights into the effectiveness of the IMU-based method for detecting gait abnormalities. Second, only male subjects participated in this experiment, making it difficult to generalize the study results to all genders. Moreover, this study focused solely on healthy participants, which did not fully capture the complexities of gait abnormalities experienced by actual patients. Additionally, this study was conducted in a controlled laboratory environment, limiting the applicability of the results to real-world settings. Therefore, in future research, we plan to recruit a larger number of participants from an expanded pool that considers factors such as gender, age, and medical conditions to enhance the reliability and generalizability of the results. Furthermore, the experimental protocol will be designed to consider real-world walking surfaces, speeds, and individual gait characteristics. Comprehensive data will be collected from patients with various types and severities of abnormal gait. Finally, by monitoring changes in gait patterns over time-related to rehabilitation and disease progression, we aim to develop abnormal gait classification algorithms optimized for diverse environmental conditions and patient types.

## 5. Conclusions

This study utilized various classification models to differentiate walking characteristics under different walking conditions, focusing on knee and ankle joint impairments. By employing the IMU-based system and walkway, we evaluated the performance of these models in analyzing walking data collected from the same subjects. Statistical analyses were conducted to scrutinize kinematic data, and experiments were designed to induce changes in walking conditions among the participants.

The results indicate that the IMU-based system outperformed the walkway in all classification tasks, particularly demonstrating significant disparities in two-class classification tasks between knee and ankle joint impairments. Features such as the knee sagittal angle, shank sagittal angle, swing/stance time, swing phase, double support time, and TSR were identified as important in distinguishing between ankle and knee impairments, highlighting the superiority of an IMU-based system in capturing relevant walking characteristics.

This study provides valuable insights into how various impairments impact walking behavior, offering implications for diagnosing and managing a range of conditions, from orthopedic ailments to neurological disorders. By discerning subtle changes in walking patterns, early indicators of skeletal or neurological issues can potentially be identified, leading to tailored interventions and improved patient outcomes.

In conclusion, our study demonstrates the superior performance of the IMU-based system in classifying abnormal gait patterns with joint impairments. The precise measurement of angular parameters enables more accurate detection of specific joint impairments, surpassing the capabilities of spatio-temporal parameters alone. Future research could investigate the potential for real-time gait assessment and intervention using IMU sensors and advanced machine learning techniques.

## Figures and Tables

**Table 1:** Participant characteristics.

Participant	Age (Years)	Height (cm)	Weight (kg)
1	30	176	93
2	59	168	72
3	33	188	82
4	43	177	65
5	35	178	73
6	53	165	65
7	46	176	65
8	27	174	95
9	28	181	100
10	40	173	80

**Table 2:** Extracted gait parameters.

System	Categories	Parameters
IMU-based	Angular	shank sagittal angle (deg), thigh sagittal angle (deg), knee sagittal angle (deg)
	Spatio-temporal	cycle time (s), swing time (s), stance time (s), swing phase, stance phase, cadence (steps/min)
	Symmetry	SSR 1, SSR 2, TSR
Walkway	Spatio-temporal	step length (cm), stride length (cm), base of support (cm), step time (s), stride time (s), swing time (s), stance time (s), single support time (s), double support time (s), stride velocity (cm/s), swing phase, stance phase
	Symmetry	TSR

**Table 3:** The average results of the cross-validation for each model.

System	Types	Classifier	Optimal Number of Features	Accuracy	Precision	Recall	F1 Score
IMU-based	normal -abnormal	SVM	1	0.88	0.90	0.88	0.88
		RF	12	0.99	0.99	0.99	0.99
		XGB	5	0.96	0.97	0.96	0.96
	normal -knee	SVM	1	1.00	1.00	1.00	1.00
		RF	1	1.00	1.00	1.00	1.00
		XGB	1	0.99	1.00	0.99	0.99
	normal -ankle	SVM	1	0.87	0.90	0.87	0.87
		RF	2	0.97	0.98	0.97	0.97
		XGB	7	0.93	0.94	0.93	0.93
	knee -ankle	SVM	5	0.97	0.97	0.97	0.97
		RF	18	0.98	0.98	0.98	0.98
		XGB	3	0.98	0.98	0.98	0.98
	normal -knee -ankle	SVM	6	0.90	0.90	0.90	0.90
		RF	11	0.91	0.91	0.91	0.91
		XGB	5	0.89	0.90	0.89	0.89
Walkway	normal -abnormal	SVM	1	0.90	0.92	0.90	0.91
		RF	15	0.90	0.90	0.90	0.90
		XGB	12	0.90	0.90	0.90	0.90
	normal -knee	SVM	1	0.92	0.93	0.92	0.92
		RF	3	0.94	0.94	0.94	0.93
		XGB	2	0.93	0.93	0.93	0.93
	normal -ankle	SVM	12	0.91	0.91	0.91	0.91
		RF	4	0.91	0.91	0.91	0.90
		XGB	4	0.90	0.90	0.90	0.90
	knee -ankle	SVM	9	0.76	0.76	0.76	0.76
		RF	19	0.75	0.75	0.75	0.74
		XGB	10	0.75	0.75	0.75	0.75
	normal -knee -ankle	SVM	9	0.73	0.73	0.73	0.73
		RF	10	0.75	0.75	0.75	0.75
		XGB	21	0.77	0.77	0.77	0.77

**Table 4:** Optimal features across models.

Types	IMU-Based System Features	Walkway System Features
normal-abnormal	TSR	TSR
normal-knee	Knee sagittal angle (R)	TSR
normal-ankle		Swing time (R)
knee-ankle	Shank sagittal angle (R)	Stance time (L), stance time (R), swing phase (L), swing phase (R), double support time (L), double support time (R)
normal-knee-ankle	Knee sagittal angle (R)	TSR, swing phase (R), double support time (R)

**Table 5:** Five-fold cross-validation performance metrics on the highest-performing classifiers.

System	Classifier	Fold	Accuracy	Precision	Recall	F1 Score
IMU-based	RF	1	0.93	0.94	0.93	0.94
		2	0.97	0.97	0.97	0.97
		3	0.83	0.88	0.83	0.86
		4	0.95	0.96	0.95	0.95
		5	0.85	0.90	0.85	0.87
Walkway	XGB	1	0.85	0.88	0.85	0.86
		2	0.80	0.80	0.80	0.80
		3	0.68	0.81	0.68	0.74
		4	0.75	0.81	0.75	0.78
		5	0.75	0.75	0.75	0.75

**Table 6:** Descriptive statistics and Friedman test results for gait in the knee and ankle joint impairment classification.

System	Feature	Side	Normal	Knee	Ankle	Friedman	
						Statistic	*p*-Value
IMU-based	Shank sagittal angle (deg)	L	68.51 ± 5.06	63.98 ± 5.36	59.51 ± 5.58	16.8	0.0002
		R	66.99 ± 5.0	47.66 ± 5.33	51.14 ± 12.3	12.6	0.0018
	Thigh sagittal angle (deg)	L	35.27 ± 4.31	34.24 ± 3.25	34.3 ± 3.9	0.2	0.9048
		R	36.26 ± 4.72	38.96 ± 4.22	37.18 ± 5.7	2.4	0.3012
	Knee sagittal angle (deg)	L	57.57 ± 5.87	53.74 ± 5.95	49.77 ± 6.6	16.8	0.0002
		R	55.63 ± 6.97	12.81 ± 4.88	36.68 ± 14.58	18.2	0.0001
	Cycle time (s)	L	1.12 ± 0.04	1.25 ± 0.12	1.33 ± 0.12	11.4	0.0033
		R	1.12 ± 0.05	1.25 ± 0.12	1.33 ± 0.13	12.8	0.0017
	Cadence (steps/min)	L	53.83 ± 2.13	48.53 ± 4.61	45.4 ± 4.33	11.4	0.0033
		R	53.71 ± 2.35	48.57 ± 4.67	45.41 ± 4.45	12.8	0.0017
	Swing time (s)	L	0.49 ± 0.03	0.49 ± 0.03	0.48 ± 0.04	0.6	0.7408
		R	0.49 ± 0.02	0.61 ± 0.07	0.59 ± 0.06	15.2	0.0005
	Stance time (s)	L	0.63 ± 0.03	0.76 ± 0.11	0.86 ± 0.1	14.6	0.0007
		R	0.63 ± 0.04	0.64 ± 0.06	0.74 ± 0.08	15.0	0.0006
	Swing phase	L	0.44 ± 0.02	0.4 ± 0.03	0.36 ± 0.03	16.8	0.0002
		R	0.44 ± 0.01	0.49 ± 0.03	0.44 ± 0.03	15.8	0.0004
	Stance phase	L	0.56 ± 0.02	0.6 ± 0.03	0.64 ± 0.03	16.8	0.0002
		R	0.56 ± 0.01	0.51 ± 0.03	0.56 ± 0.03	15.8	0.0004
	SSR 1		0.96 ± 0.1	0.66 ± 0.07	0.79 ± 0.14	14.6	0.0007
	SSR 2		0.98 ± 0.15	0.24 ± 0.08	0.75 ± 0.34	18.2	0.0001
	TSR		1.0 ± 0.08	1.49 ± 0.29	1.45 ± 0.28	15.2	0.0005
Walkway	Step length (cm)	L	63.89 ± 4.26	56.56 ± 5.16	49.28 ± 8.49	18.2	0.0001
		R	63.67 ± 4.55	57.63 ± 4.72	55.48 ± 5.65	9.8	0.0074
	Stride length (cm)	L	128.04 ± 8.89	114.89 ± 8.48	105.4 ± 12.37	16.8	0.0002
		R	128.05 ± 8.72	114.3 ± 8.54	104.7 ± 12.27	16.8	0.0002
	Base of support (cm)	L	11.59 ± 3.14	17.59 ± 4.28	18.83 ± 3.32	15.2	0.0005
		R	11.63 ± 3.11	17.44 ± 4.43	18.68 ± 3.23	15.2	0.0005
	Step time (s)	L	0.57 ± 0.03	0.59 ± 0.04	0.63 ± 0.07	9.8	0.0074
		R	0.57 ± 0.03	0.67 ± 0.08	0.72 ± 0.08	16.8	0.0002
	Stride time (s)	L	1.13 ± 0.05	1.25 ± 0.12	1.35 ± 0.13	12.8	0.0017
		R	1.13 ± 0.05	1.26 ± 0.12	1.35 ± 0.13	12.8	0.0017
	Stride velocity (cm/s)	L	113.75 ± 9.98	92.89 ± 14.27	79.11 ± 13.07	16.8	0.0002
		R	113.95 ± 10.01	92.09 ± 14.4	78.56 ± 13.12	16.8	0.0002
	Double support time (s)	L	0.26 ± 0.02	0.29 ± 0.05	0.38 ± 0.06	16.8	0.0002
		R	0.26 ± 0.03	0.29 ± 0.05	0.38 ± 0.06	16.8	0.0002
	Single support time (s)	L	0.43 ± 0.02	0.54 ± 0.07	0.54 ± 0.06	15.2	0.0005
		R	0.43 ± 0.02	0.43 ± 0.03	0.43 ± 0.05	0.6	0.7408
	Swing time (s)	L	0.43 ± 0.02	0.43 ± 0.03	0.43 ± 0.05	0.6	0.7408
		R	0.43 ± 0.02	0.54 ± 0.07	0.54 ± 0.06	15.2	0.0005
	Stance time (s)	L	0.7 ± 0.04	0.82 ± 0.11	0.92 ± 0.1	12.8	0.0017
		R	0.7 ± 0.04	0.72 ± 0.06	0.8 ± 0.09	13.4	0.0012
	Swing phase	L	0.38 ± 0.01	0.35 ± 0.03	0.32 ± 0.03	16.8	0.0002
		R	0.38 ± 0.01	0.42 ± 0.02	0.4 ± 0.03	11.4	0.0033
	Stance phase	L	0.62 ± 0.01	0.65 ± 0.03	0.68 ± 0.03	16.8	0.0002
		R	0.62 ± 0.01	0.58 ± 0.02	0.6 ± 0.03	11.4	0.0033
	TSR		1.0 ± 0.06	1.41 ± 0.23	1.5 ± 0.33	15.8	0.0004

## Data Availability

The data used in this study are available on request from the corresponding author. The data are not publicly available because of participant confidentiality.
